# Supplementary material for: The barriers and enablers of outbreak reporting in the Asia-Pacific region: A mixed methods study of field epidemiologists
Source: PLOS Glob Public Health. 2026 Jan 8;6(1):e0005640. doi: 10.1371/journal.pgph.0005640 (PMC12782425; doi:10.1371/journal.pgph.0005640)
Supplement: S2 Text — (PDF) [file pgph.0005640.s002.pdf]

Survey on barriers and enablers of outbreak reporting.

Outbreak reporting

Start of Block: Informed Consent

Q1 The National Centre for Epidemiology and Population Health at the Australian National University is conducting research on outbreak reporting. Outbreak reporting is defined here as *the reporting of the presence or suspected presence of an infectious disease outbreak to or between public health officials at the local, national, or international level*.

We are conducting a survey on the **outbreak reporting barriers (things that make reporting more difficult) and enablers (things that make reporting easier)** that are experienced or observed by **trainees and graduates of Field Epidemiology Training Programs (FETPs) in the World Health Organization (WHO) South-East Asia and Western Pacific Regions**.

This survey will take about 15 minutes to complete. Your responses are anonymous (unless you willingly provide your contact information at the end of the survey), and you can choose not to answer any questions you are not comfortable answering by selecting "Prefer not to say".

At the end of each survey section, you will be asked if you would you like to continue the survey or withdraw from it. If you elect to withdraw from the survey, you will be asked to confirm your response. Once you confirm your response, you will be withdrawn from the survey. <u>None of your responses will be recorded if you choose to withdraw.</u>

Before beginning this survey, we ask that you review the Participant Information Sheet linked below. After reading it, please indicate your consent below to start the survey. Thank you for your participation.

Q2 Click to write the question text

Q3 I have read and understood the Participant Information Sheet, and I consent to participate in this research.

1. Yes (1)
2. No (2)

End of Block: Informed Consent

Start of Block: FETP

Q4 In the first section, we will ask you questions about your education, FETP experience, professional background, and public health work.

Q5 Are you a current Field Epidemiology Training Program (FETP) trainee, or have you graduated from an FETP?

3. Current trainee (1)
4. Graduate (2)
5. Neither current trainee nor graduate (4)

End of Block: FETP

Start of Block: Background questions

Q6 What is the highest level of FETP that you are participating in or have completed?

6. FETP-Advanced (1)
7. FETP-Intermediate (2)
8. FETP-Frontline or Basic (3)
9. FETP for Veterinarians (6)
10. FETP (not classified or not listed above) (4)
11. Prefer not to say (5)

Display This Question:

If What is the highest level of FETP that you are participating in or have completed? = FETP (not classified or not listed above)

Q7 If your FETP level is not classified or listed in the above question, please indicate it below.

____

Q8 What year did you graduate (or will graduate) from the highest level of FETP you are participating in or have completed? Please specify the full year (for example, 1981).

____

Q9 Through which country did you last complete (or are currently undertaking) FETP training?

12. American Samoa (United States) (136)
13. Australia (137)
14. Bangladesh (138)
15. Bhutan (139)
16. Cambodia (140)
17. China (141)
18. Cook Islands (142)
19. Federated States of Micronesia (143)
20. Fiji (144)
21. French Polynesia (France) (145)
22. Guam (United States) (146)
23. Hong Kong (147)
24. India (148)
25. Indonesia (149)
26. Japan (150)
27. Kiribati (151)
28. Lao People's Democratic Republic (152)
29. Malaysia (153)
30. Marshall Islands (154)
31. Mongolia (155)
32. Myanmar (156)
33. Nauru (157)
34. Nepal (158)
35. New Caledonia (France) (159)
36. Niue (160)
37. Northern Mariana Islands (United States) (161)
38. Palau (162)
39. Papua New Guinea (163)
40. Philippines (164)
41. Republic of Korea (South Korea) (165)
42. Samoa (166)
43. Singapore (167)
44. Solomon Islands (168)
45. Taiwan (169)
46. Thailand (170)
47. Tokelau (New Zealand) (171)
48. Tonga (172)
49. Tuvalu (173)
50. Vanuatu (174)
51. Viet Nam (175)
52. Wallis and Futuna (France) (176)
53. Other (177)
54. Prefer not to say (178)

Display This Question:

If Through which country did you last complete (or are currently undertaking) FETP training? = Other

Q10 If you answered "Other" above, please state the country in which you last completed (or are currently undertaking) FETP training.

____

Q11 In what setting do you currently work? Choose all that apply.

1. Government public health office (42)
2. Academia/university (43)
3. Hospital or clinic (44)
4. Laboratory (45)
5. Private sector (46)
6. Non-governmental or international organization (for example, World Health Organization) (47)
7. Other (48)
8. Prefer not to say (49)

Display This Question:

If In what setting do you currently work? Choose all that apply. = Government public health office

Q12 If you answered "Government public health office" above, please indicate the type of office.

9. National health ministry/department (1)
10. State/provincial health office (2)
11. Local/district health office (3)
12. Prefer not to say (4)

Display This Question:

If In what setting do you currently work? Choose all that apply. = Other

Q13 If you answered "Other" above, please state the work you perform.

____

Q14 What is your current job title?

____

Q15 How many years have you worked in a public health role (including FETP training)?

13. Less than 5 years (12)
14. 5-9 years (13)
15. 10-14 years (14)
16. 15 years or more (15)
17. Prefer not to say (16)

Q16 Do you currently work in a public health role?

18. Yes (12)
19. No (13)
20. Prefer not to say (17)

Q17 Please indicate the country (or countries) in which you currently work. Select all that apply.

Note: if you live in one country, but your work takes place in another country (or countries), indicate

the country (or countries) where your work takes place.

21. American Samoa (United States) (915)
22. Australia (916)
23. Bangladesh (917)
24. Bhutan (918)
25. Cambodia (919)
26. China (920)
27. Cook Islands (921)
28. Federated States of Micronesia (922)
29. Fiji (923)
30. French Polynesia (France) (924)
31. Guam (United States) (925)
32. Hong Kong (926)
33. India (927)
34. Indonesia (928)
35. Japan (929)
36. Kiribati (930)
37. Lao People's Democratic Republic (931)
38. Malaysia (932)
39. Marshall Islands (933)
40. Mongolia (934)
41. Myanmar (935)
42. Nauru (936)
43. Nepal (937)
44. New Caledonia (France) (938)
45. Niue (939)
46. Northern Mariana Islands (United States) (940)
47. Palau (941)
48. Papua New Guinea (942)
49. Philippines (943)
50. Republic of Korea (South Korea) (944)
51. Samoa (945)
52. Singapore (946)
53. Solomon Islands (947)
54. Taiwan (948)
55. Thailand (949)
56. Tokelau (New Zealand) (950)
57. Tonga (951)
58. Tuvalu (952)
59. Vanuatu (953)
60. Viet Nam (954)
61. Wallis and Futuna (France) (955)
62. Other (956)
63. Prefer not to say (957)

Display This Question:

If Please indicate the country (or countries) in which you currently work. Select all that apply. No... = Other

Q18 If you answered "Other" above, please indicate in which other country (or countries) you currently work.

____

Display This Question:

If If Please indicate the country (or countries) in which you currently work. Select all that apply.Note: if you live in one country, but your work takes place in another country (or countries), indicate... q://QID1/SelectedChoicesCount Is Greater Than 1

Carry Forward Selected Choices from "Please indicate the country (or countries) in which you currently work. Select all that apply.Note: if you live in one country, but your work takes place in another country (or countries), indicate the country (or countries) where your work takes place."

Q19 If you indicated more than one country above, please indicate in which country you currently work the most.

64. Prefer not to say (1)
65. American Samoa (United States) (2)
66. Australia (3)
67. Bangladesh (4)
68. Bhutan (5)
69. Cambodia (6)
70. China (7)
71. Cook Islands (8)
72. Federated States of Micronesia (9)
73. Fiji (10)
74. French Polynesia (France) (11)
75. Guam (United States) (12)
76. Hong Kong (13)
77. India (14)
78. Indonesia (15)
79. Japan (16)
80. Kiribati (17)
81. Lao People's Democratic Republic (18)
82. Malaysia (19)
83. Marshall Islands (20)
84. Mongolia (21)
85. Myanmar (22)
86. Nauru (23)
87. Nepal (24)
88. New Caledonia (France) (25)
89. Niue (26)
90. Northern Mariana Islands (United States) (27)
91. Palau (28)
92. Papua New Guinea (29)
93. Philippines (30)
94. Republic of Korea (South Korea) (31)
95. Samoa (32)
96. Singapore (33)
97. Solomon Islands (34)
98. Taiwan (35)
99. Thailand (36)
100. Tokelau (New Zealand) (37)
101. Tonga (38)
102. Tuvalu (39)
103. Vanuatu (40)
104. Viet Nam (41)
105. Wallis and Futuna (France) (42)
106. Other (43)
107. Prefer not to say (44)

Display This Question:

If If If you indicated more than one country above, please indicate in which country you currently work... Other Is Selected

Q20 If you answered "Other" above, please indicate in which other country you currently work the most.

____

Q21 Are you responsible for reporting outbreaks as part of your work?

108. Yes (3)
109. No (4)
110. Prefer not to say (5)

Skip To: Q23 If Are you responsible for reporting outbreaks as part of your work? = No

Display This Question:

If Are you responsible for reporting outbreaks as part of your work? = Yes

Q22 If you answered yes to the above question, to whom do you report outbreaks?

____

Q23 Have you reported an outbreak in the last five years?

111. Yes (3)
112. No (4)
113. Prefer not to say (5)

Display This Question:

If Have you reported an outbreak in the last five years? = Yes

Q24 If you answered yes to the above question, how many outbreaks have you reported in the last five years?

114. 1-4 outbreaks (1)
115. 5-9 outbreaks (2)
116. 10 or more outbreaks (3)
117. Prefer not to say (4)

Q25 Do you anticipate reporting an outbreak (or outbreaks) in the coming year?

118. Yes (1)
119. No (2)
120. Prefer not to say (3)

Q26 **Would you like to continue this survey?**

121. Yes, I would like to continue this survey (2)
122. No, please withdraw me from this survey and delete my responses (1)

Display This Question:

If Would you like to continue this survey? = No, please withdraw me from this survey and delete my responses

Q27 **Are you sure you would like to withdraw from this survey?**

123. No, I chose to withdraw by mistake and would like to continue the survey (1)
124. Yes, please withdraw me from this survey and delete my responses (2)

Display This Question:

If Would you like to continue this survey? = Yes, I would like to continue this survey

Or Are you sure you would like to withdraw from this survey? = No, I chose to withdraw by mistake and would like to continue the survey

Q28 **You have reached the end of this section. To move to the next section, click the Next (right arrow) button. After you click the Next button, you will not be able to return to this section.**

End of Block: Background questions

Start of Block: Outbreak reporting barriers

Q29 In the following two sections, we will ask questions about the barriers and enablers that affect outbreak reporting in the country in which you work (or work the most).

First, we would like to hear about **your observations or experiences with outbreak reporting barriers (things that make reporting more difficult)** in your **current or recent work (within the last five years)**.

JS

Q30 Using the options below, please rate how each of the following barriers (things that make reporting more difficult) impacts outbreak reporting.

For the following statements, please respond based on what you have experienced or observed on an individual or team level in the country in which you work (or work the most). If you do not know or

are unsure about how an option might affect outbreak reporting, click "I am not sure".

	No impact (1)	Some impact (2)	High impact (3)	I am not sure (7)	Prefer not to say (8)
Staff too busy to report an outbreak. (53)	125.	126.	127.	128.	129.
Not enough available staff to report an outbreak. (54)	130.	131.	132.	133.	134.
Reporting is too complicated, difficult, or time-consuming. (55)	135.	136.	137.	138.	139.
Staff do not know what requires reporting. (56)	140.	141.	142.	143.	144.
Staff do not know how to report an outbreak. (57)	145.	146.	147.	148.	149.
Staff do not know to whom to report an outbreak. (58)	150.	151.	152.	153.	154.
Staff not motivated to report outbreaks. (59)	155.	156.	157.	158.	159.
Staff lack authority to report outbreaks. (60)	160.	161.	162.	163.	164.
Staff afraid of being punished for reporting outbreaks or being blamed for the outbreak. (61)	165.	166.	167.	168.	169.
Staff pressured to not report outbreaks. (62)	170.	171.	172.	173.	174.

Q31 Please provide any explanations or comments to help us understand the answers you provided.

____

____

____

____

____

JS

Q32 *For the following statements, please respond based on what you have experienced or observed* <u>*across the country as a whole*</u> *for the country in which you work (or work the most). If you do not know or are unsure about how an option might affect outbreak reporting, click "I am not sure".*

	No impact (1)	Some impact (2)	High impact (3)	I am not sure (6)	Prefer not to say (7)
Lack of surveillance resources to detect an outbreak. (46)	175.	176.	177.	178.	179.
Lack of laboratory resources to identify outbreak pathogen. (47)	180.	181.	182.	183.	184.
Lack of resources to report an outbreak (for example: access to telephone, computer, internet, appropriate forms). (48)	185.	186.	187.	188.	189.
Lack of reporting mandate, regulations, or legislation. (49)	190.	191.	192.	193.	194.
Government not interested in encouraging outbreak reporting or making outbreak reporting easier. (50)	195.	196.	197.	198.	199.
Difficulty coordinating with other agencies, ministries, or sectors. (51)	200.	201.	202.	203.	204.
Fear of economic damages from reporting (for example: losses to trade or tourism). (52)	205.	206.	207.	208.	209.
Fear of media exposure following reporting. (53)	210.	211.	212.	213.	214.

Concerns about protecting patient privacy. (54)	215.	216.	217.	218.	219.

Q33 Please provide any explanations or comments to help us understand the answers you provided.

____

____

____

____

____

Q34 Please describe any additional outbreak reporting barriers (things that make reporting more difficult) that you have experienced or observed that are not covered above.

____

____

____

____

____

Q35 **Would you like to continue this survey?**

220. Yes, I would like to continue this survey (2)
221. No, please withdraw me from this survey and delete my responses (1)

Display This Question:

If Would you like to continue this survey? = No, please withdraw me from this survey and delete my responses

Q36 **Are you sure you would like to withdraw from this survey?**

222. No, I chose to withdraw by mistake and would like to continue the survey (1)
223. Yes, please withdraw me from this survey and delete my responses (2)

Display This Question:

If Would you like to continue this survey? = Yes, I would like to continue this survey

Or Are you sure you would like to withdraw from this survey? = No, I chose to withdraw by mistake and would like to continue the survey

Q37 **You have reached the end of this section. To move to the next section, click the Next (right arrow) button. After you click the Next button, you will not be able to return to this section.**

End of Block: Outbreak reporting barriers

Start of Block: Outbreak reporting enablers

Q38 Next, we would like to hear about **your observations or experiences with outbreak reporting enablers (things that make reporting easier)** in your **current or recent work (within the last five years)**.

JS

Q39 Using the options below, please rate how each of the following enables outbreak reporting (makes reporting easier).

For the following statements, please respond based on what you have experienced or observed on an individual or team level in the country in which you work (or work the most). If you do not know or

are unsure about how an option might affect outbreak reporting, click "I am not sure".

	No impact (1)	Some impact (2)	High impact (3)	I am not sure (6)	Prefer not to say (7)
Easy ways to report outbreaks (for example, simplified or electronic reporting). (30)	224.	225.	226.	227.	228.
Designated person(s) responsible for reporting an outbreak. (31)	229.	230.	231.	232.	233.
Specific training about what to report and how to report an outbreak. (32)	234.	235.	236.	237.	238.
Instruction on the importance of reporting an outbreak. (33)	239.	240.	241.	242.	243.
Encouragement to report from more senior official(s). (34)	244.	245.	246.	247.	248.
Sufficient authority to report an outbreak. (35)	249.	250.	251.	252.	253.
Reimbursing or rewarding persons who report outbreaks. (36)	254.	255.	256.	257.	258.
Punishing persons who fail to report outbreaks. (37)	259.	260.	261.	262.	263.

Q40 Please provide any explanations or comments to help us understand the answers you provided.

____

____

JS

Q41 *For the following statements, please respond based on what you have experienced or observed across the country as a whole for the country in which you work (or work the most). If you do not know or are unsure about how an option might affect outbreak reporting, click "I am not sure".*

	No impact (1)	Some impact (2)	High impact (3)	I am not sure (6)	Prefer not to say (7)
Sufficient surveillance resources to detect an outbreak. (50)	264.	265.	266.	267.	268.
Sufficient laboratory resources to identify outbreak pathogen. (51)	269.	270.	271.	272.	273.
Feedback on report quality (including information on what was reported well and how reports can be improved). (52)	274.	275.	276.	277.	278.
Feedback on outbreaks reported (including epidemiological and outbreak response information). (53)	279.	280.	281.	282.	283.
Presence of reporting mandate, regulations, or legislation. (54)	284.	285.	286.	287.	288.
Government interest in encouraging outbreak reporting or making outbreak reporting easier. (55)	289.	290.	291.	292.	293.
Good coordination between other agencies, ministries, or sectors. (56)	294.	295.	296.	297.	298.

Actions to protect patient privacy (for example: promising not to share patient data or promising to destroy patient data after period of time). (57)	299.	300.	301.	302.	303.

Q42 Please provide any explanations or comments to help us understand the answers you provided.

____

____

____

____

____

Q43 Please describe any additional outbreak reporting enablers (things that make reporting easier) that you have experienced or observed that are not covered above.

____

____

____

____

____

Q44 **Would you like to continue this survey?**

304. Yes, I would like to continue this survey (2)
305. No, please withdraw me from this survey and delete my responses (1)

Display This Question:

If Would you like to continue this survey? = No, please withdraw me from this survey and delete my responses

Q45 **Are you sure you would like to withdraw from this survey?**

306. No, I chose to withdraw by mistake and would like to continue the survey (1)
307. Yes, please withdraw me from this survey and delete my responses (2)

Display This Question:

If Would you like to continue this survey? = Yes, I would like to continue this survey

Or Are you sure you would like to withdraw from this survey? = No, I chose to withdraw by mistake and would like to continue the survey

Q46 **You have reached the end of this section. To move to the next section, click the Next (right arrow) button. After you click the Next button, you will not be able to return to this section.**

End of Block: Outbreak reporting enablers

Start of Block: Demographics

Q47 In this final section, the following questions about you are important to help us interpret the results of the survey. If there are any questions you would rather not answer, please select "Prefer not to say".

Q48 What is your age?

308. 24 years or younger (1)
309. 25-34 years (2)
310. 35-44 years (3)
311. 45-54 years (4)
312. 55-64 years (5)
313. 65 years or older (6)
314. Prefer not to say (7)

Q49 With which gender identity do you most identify?

315. Male (1)
316. Female (2)
317. Non-binary / third gender (3)
318. Prefer not to say (4)

Q50 What is the highest level of education you have completed?

319. Some secondary school or less (13)
320. Completed secondary school (14)
321. Trade/technical/vocational training (15)
322. Associate or Bachelor's degree (not including medical or veterinary degree) (16)
323. Postgraduate certificate or diploma (17)
324. Master's degree (18)
325. Clinical degree (including medical or veterinary degree) (19)
326. Doctorate degree (including PhD) (20)
327. Other (21)
328. Prefer not to say (22)

Display This Question:

If What is the highest level of education you have completed? = Other

Q51 If you answered "Other" above, please indicate your highest level of education.

____

Q52 If you have any comments relating to this survey, please provide them below.

____

____

____

____

____

Q53 **Would you like to continue this survey?**

329. Yes, I would like to continue this survey (2)
330. No, please withdraw me from this survey and delete my responses (1)

Display This Question:

If Would you like to continue this survey? = No, please withdraw me from this survey and delete my responses

Q54 **Are you sure you would like to withdraw from this survey?**

331. No, I chose to withdraw by mistake and would like to continue the survey (1)
332. Yes, please withdraw me from this survey and delete my responses (2)

Display This Question:

If Would you like to continue this survey? = Yes, I would like to continue this survey

Or Are you sure you would like to withdraw from this survey? = No, I chose to withdraw by mistake and would like to continue the survey

Q55 **You have reached the end of this section. To move to the next section, click the Next (right arrow) button. After you click the Next button, you will not be able to return to this section.**

End of Block: Demographics

Start of Block: Follow-up question

Q56 **Follow-up Research**

We plan to interview a select number of respondents to this survey to gather more in-depth insights into the responses provided. The information you provide in the interview will be de-identified (references to your name or identity will be removed) to protect your privacy. Are you interested in being interviewed?

333. Yes (1)
334. No (2)

Display This Question:

If Follow-up Research We plan to interview a select number of respondents to this survey to gather m... = Yes

Q57 If you are interested in being interviewed by one of our researchers, please provide your name and email address below.

335. First name (1) ____

336. Last name (2) ____

337. Email address (3) ____

End of Block: Follow-up question

Start of Block: Submit survey

Q58 **Thank you for completing this survey. Please click the Next (right arrow) button below to submit it.**

Q59 ***This is your final opportunity to withdraw from this survey and delete your responses. After you hit the Next (right arrow) button, your responses will be submitted.***

Would you like to submit this survey?

338. Yes, I would like to submit this survey (2)

339. No, please withdraw me from this survey and delete my responses (1)

Display This Question:

If This is your final opportunity to withdraw from this survey and delete your responses. After you... = No, please withdraw me from this survey and delete my responses

Q60 **Are you sure you would like to withdraw from this survey?**

340. No, I chose to withdraw by mistake and would like to submit this survey (1)

341. Yes, please withdraw me from this survey and delete my responses (2)

End of Block: Submit survey
