# Supplementary material for: The barriers and enablers of outbreak reporting in the Asia-Pacific region: A mixed methods study of field epidemiologists
Source: PLOS Glob Public Health. 2026 Jan 8;6(1):e0005640. doi: 10.1371/journal.pgph.0005640 (PMC12782425; doi:10.1371/journal.pgph.0005640)
Supplement: S3 Text — (PDF) [file pgph.0005640.s003.pdf]

Guide for interviews on barriers and enablers of outbreak reporting.

- Can you briefly describe your background – your public/animal health training and experience?
- Are you familiar with the outbreak reporting system in your country?
    - [If yes]
        - How are outbreaks typically reported?
        - What has been your experience with the system's implementation during outbreaks?
        - Were you involved in making the decision to report outbreaks or the outbreak reporting chain?
            - [If yes]
                - What factors did you consider when deciding whether to report the outbreak?
                - Can you describe any specific things that facilitated or inhibited the outbreak reporting process?
                    - How did these affect the reporting process?
                - Several have described lack of sufficient personnel, resources, or enough time to report as barriers to reporting. Can you describe how this affects the reporting process in [country]? Examples?
                - Others have described being pressured to not report outbreaks or needing approval to report. Can you describe any specific pressures from either outside or inside your agency on the reporting process?
                    - How did these affect the reporting process? Examples?
                - Several have described having improved reporting capacity, like personnel, as a facilitator of outbreak reporting. Would this be true in your context?
                - Can you describe any other specific things that facilitated or inhibited the outbreak reporting process?
                    - How did these affect the reporting process? Examples?
                - Were any of these outbreaks reported to the World Health Organization (WHO)?
                    - Why or why not?
                - How do you feel about the reporting process itself?
            - [If no]
                - Can you describe any other specific things that facilitated or inhibited the outbreak reporting process?
                    - How did these affect the reporting process?
                - Several have described lack of sufficient personnel or resources as barriers to reporting. Can you describe how this affects the reporting process in [country]?
                - Others have described being pressured to not report outbreaks or needing approval to report. Can you describe

any specific pressures from either outside or inside your agency on the reporting process?
            - How did these affect the reporting process?
        - Can you describe any other specific things that facilitated or inhibited the outbreak reporting process?
            - How did these affect the reporting process?
        - Were any of these outbreaks reported to the World Health Organization (WHO)?
            - Why or why not? Criteria for reporting?
            - How did the reporting process go?
        - How do you feel about the reporting process itself?
    - [If no]
        - How do you imagine such a system would be implemented during outbreaks?
- Can you describe an outbreak or outbreaks that stand out with respect to how or why they were reported?
    - What makes this/these outbreak(s) stand out?
- Describe for me your vision of the ideal outbreak reporting process.
- What kind of changes or improvements do you think are necessary to implement your vision?
- What kind of barriers do you anticipate to implementing these changes?
