# Supplementary material for: The barriers and enablers of outbreak reporting in the Asia-Pacific region: A mixed methods study of field epidemiologists
Source: PLOS Glob Public Health. 2026 Jan 8;6(1):e0005640. doi: 10.1371/journal.pgph.0005640 (PMC12782425; doi:10.1371/journal.pgph.0005640)
Supplement: S4 Text — (PDF) [file pgph.0005640.s004.pdf]

Coding scheme for interviews on barriers and enablers of outbreak reporting.

- **Barriers**
  - *Behavioural barriers*
    - Lack of community involvement or cooperation
    - Lack of incentives
    - Lack of motivation to report
    - Local officials believe can handle without declaring, reporting outbreak
    - Personal consequences from reporting
      - Fear of legal consequences
      - Reluctance to report stigmatizing condition
      - Reporting as taboo - fear of punishment, career impact
        - Reporters suppress, or told by superiors to suppress, derogatory information to avoid scrutiny
    - Priority is to investigate, control then report
  - *Bureaucratic barriers*
    - Have to follow reporting hierarchy, get permission to report
    - Lack of communication, coordination among different agencies, sectors, systems
      - Private clinics failing to report
    - Lack of legislation facilitating outbreak reporting
      - Data privacy protections
  - *Lack of capacity*
    - Insufficient or overburdened personnel, lack of designated person
      - High personnel turnover
      - Personnel do not perform adequately
    - Insufficient reporting infrastructure
      - Lack of laboratory or diagnostic capacity
      - Lack of transportation infrastructure
      - Lack of utilities (power, telecommunications), IT infrastructure, electronic reporting
    - Lack of funding
    - Limited surveillance system
    - Personnel lack knowledge
      - Personnel do not know how to report appropriately
      - Personnel do not know what to report
      - Personnel do not understand that have to report, who has to report
      - Personnel lack understanding of importance of reporting
    - Poor data quality
    - Reporting guidelines are absent, inappropriate, or outdated
  - *Socioeconomic and political barriers*
    - Avoid reputational loss
      - Avoiding media limelight
      - Fear of public reaction
    - Fear of consequences for economy, trade or tourism
    - Government suppression, lack of transparency
      - Decentralization, local resistance to reporting
    - Lack of official understanding or interest
- **Enablers**
  - *Build capacity*

  - Clear, simplified reporting
    - Purpose of data is clear
  - Effective surveillance system
    - Community members can easily report
  - Improving personnel capacity
    - Designated reporter
    - Educating, training personnel
      - Personnel understand need to report
    - Experienced personnel
    - Good leadership
    - More reporting personnel
  - Reporting infrastructure
    - Adequate laboratory capacity
    - Adequate reporting forms
    - Adequate telecommunications, electronic reporting
    - Strong health system
  - Supportive financing
- *Bureaucratic support*
  - Communication, coordination with other agencies or sectors, other involved parties
  - Legal or policy authority to report
  - Official support
- *Encourage personnel to report*
  - Acknowledge or reward personnel for reporting
  - Develop reporting norms
  - Support, feedback from superiors
- *Population education about outbreak reporting*
- *Unofficial reporting*
  - Media reporting
