# Supplementary material for: The barriers and enablers of outbreak reporting in the Asia-Pacific region: A mixed methods study of field epidemiologists
Source: PLOS Glob Public Health. 2026 Jan 8;6(1):e0005640. doi: 10.1371/journal.pgph.0005640 (PMC12782425; doi:10.1371/journal.pgph.0005640)
Supplement: S1 Table — (PDF) [file pgph.0005640.s006.pdf]

Survey responses for barriers and enablers to outbreak reporting among respondents working in lower-middle-income countries (n=42).*

Category	Number of respondents (n=42)			
	High impact (%)	Some impact (%)	No impact (%)	Unsure or no answer (%)
Outbreak reporting barriers				
Individual or team-level barriers				
Staff too busy to report an outbreak	18 (42.9)	18 (42.9)	3 (7.1)	3 (7.1)
Not enough available staff to report an outbreak	22 (52.4)	11 (26.2)	7 (16.7)	2 (4.8)
Reporting is too complicated, difficult, or time-consuming	16 (38.1)	24 (57.1)	1 (2.4)	1 (2.4)
Staff do not know what requires reporting	17 (40.5)	20 (47.6)	2 (4.8)	3 (7.1)
Staff do not know how to report an outbreak	18 (42.9)	20 (47.6)	2 (4.8)	2 (4.8)
Staff do not know to whom to report an outbreak	16 (38.1)	16 (38.1)	9 (21.4)	1 (2.4)
Staff not motivated to report outbreaks	20 (47.6)	18 (42.9)	4 (9.5)	0 (0.0)
Staff lack authority to report outbreaks	17 (40.5)	17 (40.5)	7 (16.7)	1 (2.4)
Staff afraid of being punished for reporting outbreaks or being blamed for the outbreak	20 (47.6)	16 (38.1)	6 (14.3)	0 (0.0)
Staff pressured to not report outbreaks	18 (42.9)	14 (33.3)	8 (19.0)	2 (4.8)
Country-level barriers				
Lack of surveillance resources to detect an outbreak	21 (50.0)	18 (42.9)	2 (4.8)	1 (2.4)
Lack of laboratory resources to identify outbreak pathogen	22 (52.4)	14 (33.3)	4 (9.5)	2 (4.8)
Lack of resources to report an outbreak (for example: access to telephone, computer, internet, appropriate forms)	14 (33.3)	22 (52.4)	5 (11.9)	1 (2.4)
Lack of reporting mandate, regulations, or legislation	19 (45.2)	13 (31.0)	6 (14.3)	4 (9.5)

Government not interested in encouraging outbreak reporting or making outbreak reporting easier	20 (47.6)	12 (28.6)	8 (19.0)	2 (4.8)
Difficulty coordinating with other agencies, ministries, or sectors	17 (40.5)	19 (45.2)	4 (9.5)	2 (4.8)
Fear of economic damages from reporting (for example: losses to trade or tourism)	18 (42.9)	17 (40.5)	6 (14.3)	1 (2.4)
Fear of media exposure following reporting	19 (45.2)	18 (42.9)	4 (9.5)	1 (2.4)
Concerns about protecting patient privacy	7 (16.7)	22 (52.4)	12 (28.6)	1 (2.4)
Outbreak reporting enablers				
Individual or team-level enablers				
Easy ways to report outbreaks (for example, simplified or electronic reporting)	27 (64.3)	7 (16.7)	3 (7.1)	5 (11.9)
Designated persons(s) responsible for reporting an outbreak	28 (66.7)	6 (14.3)	2 (4.8)	6 (14.3)
Specific training about what to report and how to report an outbreak	31 (73.8)	6 (14.3)	1 (2.4)	4 (9.5)
Instruction on the importance of reporting an outbreak	27 (64.3)	6 (14.3)	3 (7.1)	6 (14.3)
Encouragement to report from more senior official(s)	25 (59.5)	10 (23.8)	2 (4.8)	5 (11.9)
Sufficient authority to report an outbreak	23 (54.8)	12 (28.6)	2 (4.8)	5 (11.9)
Reimbursing or rewarding persons who report outbreaks	17 (40.5)	12 (28.6)	7 (16.7)	6 (14.3)
Punishing persons who fail to report outbreaks	9 (21.4)	15 (35.7)	11 (26.2)	7 (16.7)
Country-level enablers				
Sufficient surveillance resources to detect an outbreak	32 (76.2)	6 (14.3)	1 (2.4)	3 (7.1)

Sufficient laboratory resources to identify outbreak pathogen	25 (59.5)	13 (31.0)	1 (2.4)	3 (7.1)
Feedback on report quality (including information on what was reported well and how reports can be improved)	24 (57.1)	10 (23.8)	2 (4.8)	6 (14.3)
Feedback on outbreaks reported (including epidemiological and outbreak response information)	28 (66.7)	9 (21.4)	1 (2.4)	4 (9.5)
Presence of reporting mandate, regulations, or legislation	21 (50.0)	15 (35.7)	2 (4.8)	4 (9.5)
Government interest in encouraging outbreak reporting or making outbreak reporting easier	28 (66.7)	9 (21.4)	1 (2.4)	4 (9.5)
Good coordination between other agencies, ministries, or sectors	30 (71.4)	5 (11.9)	1 (2.4)	6 (14.3)
Actions to protect patient privacy (for example: promising not to share patient data or promising to destroy patient data after period of time)	19 (45.2)	15 (35.7)	3 (7.1)	5 (11.9)

* Including one respondent who worked in lower-middle-, upper-middle-, and high-income countries.
