# Supplementary material for: The barriers and enablers of outbreak reporting in the Asia-Pacific region: A mixed methods study of field epidemiologists
Source: PLOS Glob Public Health. 2026 Jan 8;6(1):e0005640. doi: 10.1371/journal.pgph.0005640 (PMC12782425; doi:10.1371/journal.pgph.0005640)
Supplement: S3 Table — (PDF) [file pgph.0005640.s008.pdf]

Survey responses for barriers and enablers to outbreak reporting among respondents working in high-income countries (n=6).*

Category	Number of respondents (n=6)			
	High impact (%)	Some impact (%)	No impact (%)	Unsure or no answer (%)
Outbreak reporting barriers				
Individual or team-level barriers				
Staff too busy to report an outbreak	2 (33.3)	4 (66.7)	0 (0.0)	0 (0.0)
Not enough available staff to report an outbreak	2 (33.3)	3 (50.0)	0 (0.0)	1 (16.7)
Reporting is too complicated, difficult, or time-consuming	3 (50.0)	3 (50.0)	0 (0.0)	0 (0.0)
Staff do not know what requires reporting	3 (50.0)	1 (16.7)	0 (0.0)	2 (33.3)
Staff do not know how to report an outbreak	5 (83.3)	1 (16.7)	0 (0.0)	0 (0.0)
Staff do not know to whom to report an outbreak	2 (33.3)	1 (16.7)	3 (50.0)	0 (0.0)
Staff not motivated to report outbreaks	1 (16.7)	4 (66.7)	1 (16.7)	0 (0.0)
Staff lack authority to report outbreaks	2 (33.3)	1 (16.7)	2 (33.3)	1 (16.7)
Staff afraid of being punished for reporting outbreaks or being blamed for the outbreak	1 (16.7)	2 (33.3)	3 (50.0)	0 (0.0)
Staff pressured to not report outbreaks	2 (33.3)	1 (16.7)	3 (50.0)	0 (0.0)
Country-level barriers				
Lack of surveillance resources to detect an outbreak	1 (16.7)	4 (66.7)	1 (16.7)	0 (0.0)
Lack of laboratory resources to identify outbreak pathogen	3 (50.0)	2 (33.3)	1 (16.7)	0 (0.0)
Lack of resources to report an outbreak (for example: access to telephone, computer, internet, appropriate forms)	0 (0.0)	3 (50.0)	3 (50.0)	0 (0.0)
Lack of reporting mandate, regulations, or legislation	2 (33.3)	2 (33.3)	2 (33.3)	0 (0.0)

Government not interested in encouraging outbreak reporting or making outbreak reporting easier	1 (16.7)	2 (33.3)	2 (33.3)	1 (16.7)
Difficulty coordinating with other agencies, ministries, or sectors	2 (33.3)	3 (50.0)	1 (16.7)	0 (0.0)
Fear of economic damages from reporting (for example: losses to trade or tourism)	0 (0.0)	1 (16.7)	4 (66.7)	1 (16.7)
Fear of media exposure following reporting	2 (33.3)	2 (33.3)	2 (33.3)	0 (0.0)
Concerns about protecting patient privacy	1 (16.7)	3 (50.0)	2 (33.3)	0 (0.0)
Outbreak reporting enablers				
Individual or team-level enablers				
Easy ways to report outbreaks (for example, simplified or electronic reporting)	5 (83.3)	1 (16.7)	0 (0.0)	0 (0.0)
Designated persons(s) responsible for reporting an outbreak	5 (83.3)	1 (16.7)	0 (0.0)	0 (0.0)
Specific training about what to report and how to report an outbreak	6 (100.0)	0 (0.0)	0 (0.0)	0 (0.0)
Instruction on the importance of reporting an outbreak	4 (66.7)	2 (33.3)	0 (0.0)	0 (0.0)
Encouragement to report from more senior official(s)	2 (33.3)	3 (50.0)	1 (16.7)	0 (0.0)
Sufficient authority to report an outbreak	2 (33.3)	3 (50.0)	1 (16.7)	0 (0.0)
Reimbursing or rewarding persons who report outbreaks	2 (33.3)	0 (0.0)	2 (33.3)	2 (33.3)
Punishing persons who fail to report outbreaks	0 (0.0)	4 (66.7)	1 (16.7)	1 (16.7)
Country-level enablers				
Sufficient surveillance resources to detect an outbreak	5 (83.3)	1 (16.7)	0 (0.0)	0 (0.0)

Sufficient laboratory resources to identify outbreak pathogen	5 (83.3)	1 (16.7)	0 (0.0)	0 (0.0)
Feedback on report quality (including information on what was reported well and how reports can be improved)	3 (50.0)	3 (50.0)	0 (0.0)	0 (0.0)
Feedback on outbreaks reported (including epidemiological and outbreak response information)	3 (50.0)	3 (50.0)	0 (0.0)	0 (0.0)
Presence of reporting mandate, regulations, or legislation	6 (100.0)	0 (0.0)	0 (0.0)	0 (0.0)
Government interest in encouraging outbreak reporting or making outbreak reporting easier	4 (66.7)	1 (16.7)	1 (16.7)	0 (0.0)
Good coordination between other agencies, ministries, or sectors	5 (83.3)	1 (16.7)	0 (0.0)	0 (0.0)
Actions to protect patient privacy (for example: promising not to share patient data or promising to destroy patient data after period of time)	3 (50.0)	1 (16.7)	1 (16.7)	1 (16.7)

* Including one respondent who worked in lower-middle-, upper-middle-, and high-income countries.
