# Supplementary material for: The barriers and enablers of outbreak reporting in the Asia-Pacific region: A mixed methods study of field epidemiologists
Source: PLOS Glob Public Health. 2026 Jan 8;6(1):e0005640. doi: 10.1371/journal.pgph.0005640 (PMC12782425; doi:10.1371/journal.pgph.0005640)
Supplement: S1 File — Good Reporting of a Mixed Methods Study (GRAMMS) checklist. (PDF) [file pgph.0005640.s009.pdf]

Good Reporting of a Mixed Methods Study (GRAMMS) checklist

Guideline	Section
Describe the justification for using a mixed methods approach to the research question	Methods (Study Design): pg. 5
Describe the design in terms of the purpose, priority and sequence of methods	Methods (Study Design): pg. 5–6
Describe each method in terms of sampling, data collection and analysis	Methods (Study recruitment and data collection, Data analysis): pg. 6–9
Describe where integration has occurred, how it has occurred and who has participated in it	Methods (Data analysis): pg. 8–9
Describe any limitation of one method associated with the present of the other method	Discussion: pg. 20
Describe any insights gained from mixing or integrating methods	Results (Thematic barriers and enablers), Discussion: pg. 11–17, 20

Reference:

O'Cathain A, Murphy E, Nicholl J. The quality of mixed methods studies in health services research. *J Health Serv Res Policy*. 2008 Apr;13(2):92-8. doi: 10.1258/jhsrp.2007.007074.
