# Supplementary material for: The barriers and enablers of outbreak reporting in the Asia-Pacific region: A mixed methods study of field epidemiologists
Source: PLOS Glob Public Health. 2026 Jan 8;6(1):e0005640. doi: 10.1371/journal.pgph.0005640 (PMC12782425; doi:10.1371/journal.pgph.0005640)
Supplement: Attachment — Submitted filename: Response to reviewers.pdf [file pgph.0005640.s011.pdf]

PGPH-D-25-03067

The barriers and enablers of outbreak reporting in the Asia-Pacific region: a mixed methods study of field epidemiologists

PLOS Global Public Health

Re: Response to reviewers

Dear Roojin and Reviewers,

Thank you for the careful feedback regarding our manuscript. We have revised our paper based on the comments provided. Please find our responses to the comments below in italics.

1. Please include a complete copy of PLOS' questionnaire on inclusivity in global research in your revised manuscript. Our policy for research in this area aims to improve transparency in the reporting of research performed outside of researchers' own country or community. The policy applies to researchers who have travelled to a different country to conduct research, research with Indigenous populations or their lands, and research on cultural artefacts. The questionnaire can also be requested at the journal's discretion for any other submissions, even if these conditions are not met. Please find more information on the policy and a link to download a blank copy of the questionnaire here: https://journals.plos.org/globalpublichealth/s/best-practices-in-research-reporting. Please upload a completed version of your questionnaire as Supporting Information when you resubmit your manuscript.

We have provided a completed copy of the PLOS questionnaire on inclusivity in global research as Supporting Information and have updated our manuscript with a section on "Inclusivity in global research" in lines 193-195

2. Please ensure that your Ethics Statement is available in its entirety at the beginning of your Methods section, under a subheading 'Ethics Statement'.

We have renamed and moved the Ethics Statement section to the beginning of the Methods section.

3. We note that you have indicated that there are restrictions to data sharing for this study. For studies involving human research participant data or other sensitive data, we encourage authors to share de-identified or anonymized data. However, when data cannot be publicly shared for ethical reasons, we allow authors to make their data sets available upon request. For information on unacceptable data access restrictions, please see http://journals.plos.org/plosone/s/data-availability#loc-unacceptable-data-access-restrictions.

Before we proceed with your manuscript, please address the following prompts:

a) If there are ethical or legal restrictions on sharing a de-identified data set, please explain them in detail (e.g., data contain potentially identifying or sensitive patient information, data are owned by a third-party organization, etc.) and who has imposed them (e.g., a Research

Ethics Committee or Institutional Review Board, etc.). Please also provide contact information for a data access committee, ethics committee, or other institutional body to which data requests may be sent.

b) If there are no restrictions, please upload the minimal anonymized data set necessary to replicate your study findings to a stable, public repository and provide us with the relevant URLs, DOIs, or accession numbers. Please see http://www.bmj.com/content/340/bmj.c181.long for guidelines on how to de-identify and prepare clinical data for publication. For a list of recommended repositories, please see https://journals.plos.org/plosone/s/recommended-repositories. You also have the option of uploading the data as Supporting Information files, but we would recommend depositing data directly to a data repository if possible.

Please update your Data Availability statement in the submission form accordingly.

We have updated our Data Availability statement accordingly, as follows:

"Although we have taken great effort to prevent participant identification in the data used for our study, there remains a possibility of inadvertent identification. Given the highly sensitive nature of the information the participants disclosed, inadvertent identification might be particularly harmful. To protect participants from any harm related to inadvertent identification and linkage with sensitive information elicited through this study, the authors decided that all data arising from this study will be retained at the Australian National University for at least five years following publications arising from the research. After this storage period, participant data will be fully de-identified and archived at the Australian Data Archive (www.ada.edu.au) for use by other researchers. In the interim, de-identified data can be made available upon reasonable request of the corresponding author with approval of the Australian National University Human Research Ethics Committee (human.ethics.officer@anu.edu.au)."

Reviewer #1: This is an informative study on the non-technical conditions that inform reporting behaviour considerations amongst those who have the responsibility, in their national public health sectors, to detect and report suspected outbreaks.
The findings are insightful and build on/compliment existing research into the political, social and economic conditions that determine reporting behaviour.
If I had to suggest two comments to show that I read the paper (!), it would be on the conclusions that can be drawn from the sample. First, the survey was completed by those with less than 9 years of experience with a sample age quite youthful. One could suggest that reporting decisions tend to be in the remit of those with more seniority. So the vertical communication, behavioural, capacity issues and political concerns could be attributed to those with less experience and responsibility in the role, i.e. knowledge and experience will grow over time. However, a counter argument could be that the fact the 'first detectors' are identifying these limitations is a huge concern - they are the early warning mechanism. A second comment is that the sample has more provincial versus national located staff, and this could also influence the responses and findings, i.e. reporting roles and responsibility differs per country depending on their centralised/decentralised funding and regulations, which may

affect the perception of non-technical barriers. However, as I said above, these are minor comments on an excellent and informative paper that I will be citing when it is hopefully published.

You raise excellent points. We have added additional discussion about the importance of field epidemiologists' experiences around outbreak reporting barriers on the ground level and how they can address those barriers as they rise in seniority in lines 516-518 in the Discussion section. To your point about reporting roles/responsibilities differing between countries, we agree that this is an important limitation, albeit one which we believe is less impactful given the common themes that we were able to elicit through our interviews. We have added language to this effect in lines 518-521 in the Discussion section.

Reviewer #2: 1. The manuscript addresses a relevant public health question, is well structured, and presents its arguments clearly. It aligns with the journal's scope and contributes meaningfully to the literature.
2. The manuscript appears technically sound. While my training is in law rather than qualitative or quantitative methodologies, the analytical approach is coherent and the reasoning is well developed. The conclusions follow logically from the data and analysis provided.
3. The research is presented as methodologically and ethically rigorous, and the authors draw conclusions that are consistent with the evidence they provide.
4. They have not shared their data. Their data statement says that they are maintaining the data for privacy reasons. Their statement says: "To protect participants from inadvertent identification and linkage with sensitive information elicited through this study, all data arising from this study will be retained at
the Australian National University for at least five years following publications arising from the research. After this storage period, participant data will be de-identified and archived at the Australian Data Archive (www.ada.edu.au) for use by other researchers."
5. The manuscript is clearly written and well organised.

Thank you for your kind remarks. We have adopted a strict data sharing policy because of the highly sensitive nature of several of the responses. In fact, respondents have insisted on us redacting additional information during the member checking phase of this study to protect their identities, underscoring the risks they perceive from accidental identification. We have further addressed the data sharing limitations directly with the Editor, as per the above.

We hope these revisions sufficiently address the comments raised. Thank you again for the opportunity to revise and resubmit our work.

Sincerely,

Amish Talwar

Matthew M. Griffith

Rebecca Katz

Martyn D. Kirk

Tambri Housen
